# Ordinal labels in machine learning: a user-centered approach to improve data validity in medical settings

**DOI:** 10.1186/s12911-020-01152-8

**Published:** 2020-08-20

**Authors:** Andrea Seveso, Andrea Campagner, Davide Ciucci, Federico Cabitza

**Affiliations:** 1 Dipartimento di Informatica, Sistemistica e Comunicazione, Università degli Studi di Milano-Bicocca, Viale Sarca 336, Milan, 20126 Italy; 2 IRCCS Istituto Ortopedico Galeazzi, Via Riccardo Galeazzi 4, Milan, 20161 Italy

**Keywords:** Ordinal scales, Machine learning, Fuzzy sets, Ground truth

## Abstract

**Background:**

Despite the vagueness and uncertainty that is intrinsic in any medical act, interpretation and decision (including acts of data reporting and representation of relevant medical conditions), still little research has focused on how to explicitly take this uncertainty into account. In this paper, we focus on the representation of a general and wide-spread medical terminology, which is grounded on a traditional and well-established convention, to represent severity of health conditions (for instance, pain, visible signs), ranging from *Absent* to *Extreme*. Specifically, we will study how both potential patients and doctors perceive the different levels of the terminology in both quantitative and qualitative terms, and if the embedded user knowledge could improve the representation of ordinal values in the construction of machine learning models.

**Methods:**

To this aim, we conducted a questionnaire-based research study involving a relatively large sample of 1,152 potential patients and 31 clinicians to represent numerically the perceived meaning of standard and widely-applied labels to describe health conditions. Using these collected values, we then present and discuss different possible fuzzy-set based representations that address the vagueness of medical interpretation by taking into account the perceptions of domain experts. We also apply the findings of this user study to evaluate the impact of different encodings on the predictive performance of common machine learning models in regard to a real-world medical prognostic task.

**Results:**

We found significant differences in the perception of pain levels between the two user groups. We also show that the proposed encodings can improve the performances of specific classes of models, and discuss when this is the case.

**Conclusions:**

In perspective, our hope is that the proposed techniques for ordinal scale representation and ordinal encoding may be useful to the research community, and also that our methodology will be applied to other widely used ordinal scales for improving validity of datasets and bettering the results of machine learning tasks.

## Background

The machine learning community seems to put particular emphasis on performance metrics and skill improvement. And rightly so, if this general attitude has pushed some models to perform equally or even better than humans in many tasks, especially with respect to pattern recognition [1, 2].

Much smaller attention and reflection has been paid so far in regard to the validity of data, both input (training) data and output data, that is, the predictions. With validity we do not mean just accuracy, as widely intended, but above all the extent to which a measurement is well-founded and corresponds to the real world phenomena that are to be rendered in symbolic terms [3]. In other terms, we intend the *validity of a data set* as the degree to which the data set represents the phenomena it is intended to.

In order to deal with the intrinsic uncertainty of the medical domain [4], a natural choice has always been to make use of fuzzy logic and fuzzy sets. Several surveys on this connection can be found in literature, for instance [5–8]. The main use of fuzzy logic in this context is to model rules in expert systems (for example [9]) or, often in combination with other approaches such as neural networks, for image processing. On the other hand, only a few attempts to deal with the vagueness of medical terms have been made. We recall here the pioneering work to represent medical terms [10], the fuzzy version of the Arden markup language [11] and several fuzzy ontology applications to medicine [12, 13]. More related to our work is the paper [14], as discussed later in this introduction. Further, even less efforts are available on how uncertainty influences the validity of medical datasets. The recent work by Zywica [15] goes in this direction, by using fuzzy sets for transforming heterogenous data in homogenous ones and to deal with the lack of knowledge.

In this light, we set out to investigate how a specific kind of ordinal features (that is, features whose values come from a categorical label set on which an order relation is defined. In what follows, we consider these ordered categories ordinal data *natively*.) can be transformed in order to improve the *internal validity* of the training set (in the sense above), as well as the validity of the model output (that is, accuracy).

In this article we will specifically address the problem of the representation of ordinal scales in quantitative terms (and vice-versa), and the usage of these representations to define user-informed encoding to be employed in machine learning tasks, by considering the specific case of a very common terminology to represent severity of health conditions and symptoms in medical documents, which has been recently adopted also by the Health Level 7 (HL7) Fast Healthcare Interoperability Resources (FHIR) [16] framework, that is, the most widely adopted standards framework for the representation of health data on the Internet and in digital health applications [17].

This terminology is used in many questionnaires (for instance, the EQ-5D-5L [18]) aimed at collecting *Patient Reported Outcome Measures* (PROMS), which are recognized [19] as a powerful tool to enable the monitoring of the actual safety and effectiveness of medical procedures and treatments, their continuous improvement, and what is called a *value-based health care* [20, 21].

According to this terminology, both patients and doctors are called to express the severity of health conditions and symptoms in medical documents in terms of five ordinal categories, namely: *Absent* (or *No Condition*), *Mild*, *Moderate*, *Severe* and *Very Severe* (or *Extreme*) conditions. Ordinal scales are very common in medicine [22, 23] and on their basis doctors can understand each other and make critical decisions despite their seeming arbitrariness and loosely defined semantics; ordinal values like those mentioned above are also extensively used to annotate medical records, and to some extent report a written interpretation of other medical data, like laboratory results and medical images. For this reason severity labels are increasingly used in *ground truthing*, that is the preparation of training and test data sets for the definition and evaluation of predictive models. This justifies our interest in investigating whether some knowledge on how these levels are interpreted by the actors involved can affect the performance of predictive models and decision making. Although these categories are used extensively and on a daily basis by most medical doctors around the world in most forms, charts and reports (even paper-based ones), their meaning has never been established univocally and, more importantly from the computational point of view, quantitatively [24]. As a matter of fact, no standardizing body nor single doctor can establish what, say, *Moderate* really means in objective terms [25], nor determine that the transition from a *Mild* condition to a *Moderate* one is like passing from a *Moderate* one to a *Severe* condition: a standard terminology to describe severity is just a set of available values, in which only a total order relation is defined. Of course all these terms are subject to personal views, contextual situations or interpretation of evidence: in a word, they are intrinsically *fuzzy*.

More specifically, the scope of the present work is twofold:

1. Firstly, to represent severity categories using fuzzy sets by means of a collective intelligence process: by collecting the different perceptions provided by interested users, both domain experts (that is, medical doctors) and potential patients;
2. Secondly, to assess the potential impact of these techniques to construct encoding techniques for ordinal data, based on the collective knowledge, to be fed to machine learning models.

As regards the first research question, we will consider these categories as so-called linguistic labels [26] and assign them different types of fuzzy sets with domain on numerical scales according to a human-centered study. In doing so, we can get both a representative, yet approximate, model to map ordinal categories to numerical values (on a scale [0−100], where the lower bound represents absence of perceivable signs of the condition of medical interest and the upper bound its strongest expression), and *vice versa*. Also the work [14] deals with grades of questionnaire answers, however, in a different way and with a different scope with respect to us. Indeed, the aim of the authors in [14] is to define a formal logic that enables to describe the derivation of a “total” scores (typically, the average) from a set of degrees (the answers to a questionnaire). Thus, they do not address the problem of defining the total score, but, given the definition of a total score, how to describe it in a formal logic.

The data set we used to define this mapping is a collection of intervals or numerical values for each category/label, provided by both domain experts (that is, medical doctors) and potential patients by means of an ad-hoc Web-based questionnaire, administered during an online survey. We present and discuss several ways to aggregate these values in order to obtain some kind of *fuzzification* of the severity conditions.

This approach is different from existing approaches to fuzzify ordinal scales such as [27, 28], where the fuzzification process is done automatically by assigning a fuzzy number to each label and then applied to a case study. Here, our aim is to fuzzify the ordinal scale starting from the collected data and we will particularly be interested in ascertaining if the representations provided by the different respondent groups (that is doctors and potential patients) present significant differences.

As regards the second research question, the traditional approaches, adopted in the machine learning community, to deal with ordinal data in a training set [29] regard either transforming them into categorical, usually binary, values (such as one-hot encoding or rank-hot encoding), or into the rank index of the corresponding level, that is a number usually ranging from 0 to *k*.

As already introduced, we explore an alternative approach, that is encoding ordinal values in terms of scalar values on a continuous 100-point scale, according to the fuzzy set representation constructed from the subjective perceptions of the corresponding level on that scale. In doing so, we aim to embed some “true” structure into the dataset, in cases where the assumption that ordinal values are equally-distributed numbers (as in the rank index) does not hold, is ill-grounded or excessively weak.

## Methods

### Data collection

In order to build the different representations, we collected user data in three different settings, which will be discussed in this section.

#### First data collection: quantitative meaning for doctors

**Fig. 1:**
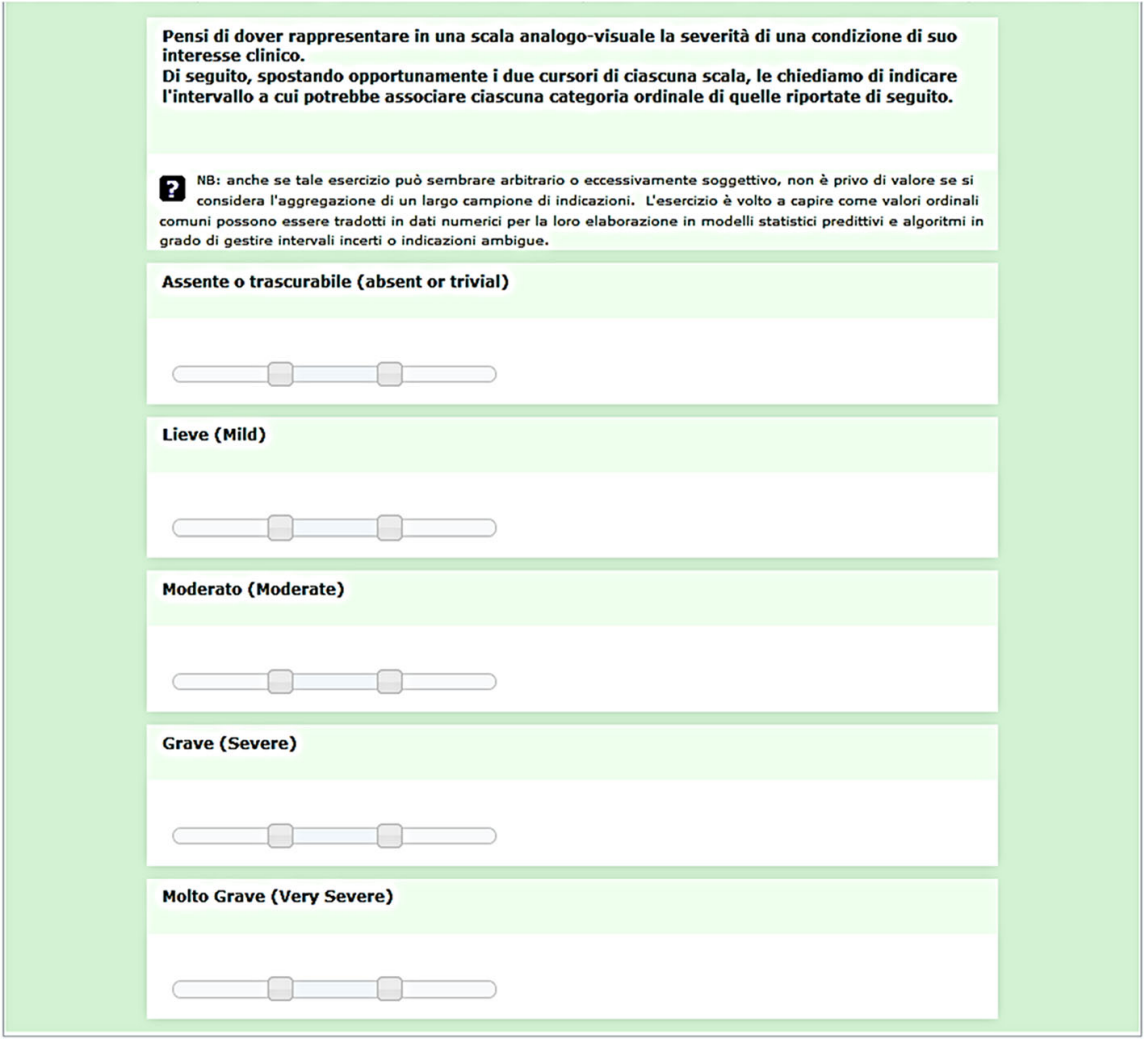
The first page of the on-line questionnaire that we administered to the sample of clinicians to collect their perception on severity categories (original text in Italian). The translation of the question asked is as follows: *“Think of having to represent the severity of a condition of clinical interest on an analogue-visual scale. Below, by appropriately moving the two cursors of each scale, we ask you to indicate the range to which each ordinal category of the following could associate”*

To collect data on the subjective perception of the quantitative meaning of the categories (each denoted by a specific label) of the severity Health Level 7 (HL7) ordinal scale, we first designed a closed-ended two-page questionnaire to be administered online in a Computer-Assisted-Web-self-Interview (CAWI) configuration. The first page of this questionnaire (depicted in Fig. 1) asked the respondents to express each level of severity of the original 5-item HL7 scale (that is, *Absent*, *Mild*, *Moderate*, *Severe*, and *Very Severe*) into a Visual Analogue Scale (VAS). A VAS is a measurement instrument that has been devised and introduced in health care to try to measure characteristics that appear or are easily perceived as continuous but that cannot be directly measured easily, like pain, and by which to overcome the intrinsically discrete nature of ordinal categorizations [30].

To this aim, we associated each item with a 2-cursor range slider control. By moving each of the two independent cursors the respondents could thus create an *inner interval*, comprised *within* the two cursors, encompassing all those numerical values that they felt could represent the ordinal category properly. The interface was designed so that initially the respondents would want to move the cursors to set the new intervals and, in doing so, “see” the overlap that they deem useful to report between the categories. This overlap was neither promoted nor prevented, as the cursors could be moved freely along each range slider with the only constraint that the ‘lower’ extreme cursor could never be moved to the right of the ‘higher’ extreme cursor, and vice versa. Moreover, the respondents could get only an approximate idea of the numerical values that were associated with the position of the cursors (and in fact this association was not mentioned in the task description, reported at the top of Fig. 1, but only in the help section), since the range was intended to be on a strict analogue scale, with no explicit nor numerical anchor. That notwithstanding, VASs are common representational tools most potential respondents were very familiar with for its wide adoption in clinical practice, as said above, and this suggests that respondents performed the task effortlessly. We also explicitly asked for a single number that the respondents could perceive as the most representative for each level: we call this number *Representative Point* (of each level, RP). The second page of the questionnaire was intended to collect a few data on the respondent’s professional profile (which was intended to be anonymous), namely their medical specialty.

**Fig. 2:**
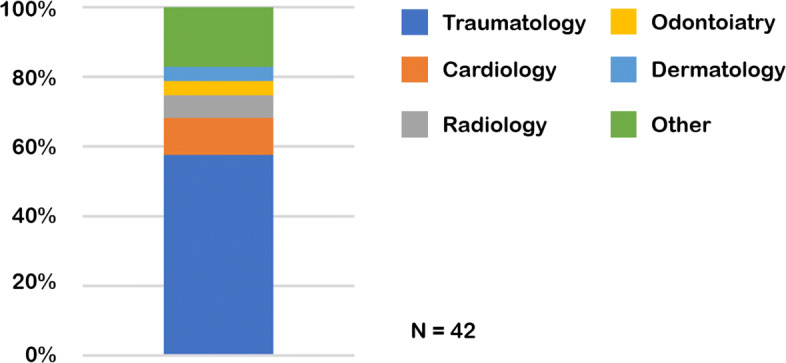
Stacked bar chart representing the composition of the sample of respondents involved in this study. The majority of the sample were trauma and orthopedic surgeons, the rest of the sample is relatively varied, as also shown by the ‘other’ category, which is the second one for numerosity and encompasses (among the others) two neurologists, one endocrinologist and one rheumatologist. This suggests that, despite the relatively small sample, this is sufficiently heterogeneous not to consider the responses limited to a specific medical discipline

**Fig. 3:**
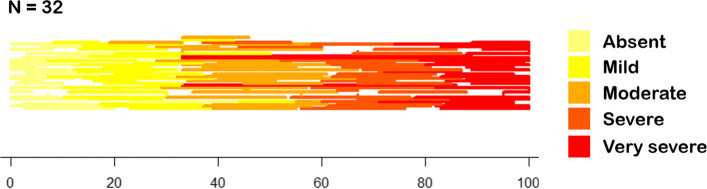
Diagram showing the data set at a glance. Different questionnaires are represented along the vertical dimension; intervals related to different severity categories are represented in different hues along the horizontal 0-100 continuum

At the end of November 2017, we invited 97 clinicians by email to fill in the two-page questionnaire. Most respondents worked as clinicians and surgeons at the Scientific Institute for Research, Hospitalization and Healthcare (IRCCS) Orthopedic Institute Galeazzi (IOG), which is one of the largest teaching hospitals in Italy specialized in the study and treatment of musculoskeletal disorders; at IOG almost 5,000 surgeries are performed yearly, mostly arthroplasty (hip and knee prosthetic surgery) and spine-related procedures. After two weeks since this first invitation we sent a gentle reminder and one week later we definitely closed the survey. Response rate was moderately high, especially in light of the very busy daily schedule of the involved prospective respondents, the anonymity of the survey and the lack of incentives: indeed slightly less than half of the potential respondents accepted the invitation and filled in the on-line questionnaire: thus we collected 42 questionnaires by as many respondents (Fig. 2). When we analyzed the responses, some questionnaires were found filled in with seemingly random data and were discarded: then the final dataset contained 298 data points, corresponding to 149 intervals (Fig. 3) by 31 different respondents. Moreover, the questionnaires completed in each and every item were 27. In doing so, we obtained an *Interval Extreme Distribution* (IED) for each severity item. The original doctor data set contained the lower and upper extremes of the five ordinal categories expressing increasing levels of severity for all of the survey respondents, that is a 31 x 10 matrix of data points on the severity dimension, ranging from 0 to 100. From this data set of coordinates of interval extremes we computed a new one, by computing the central points for each IED. An extract of this dataset is reported in Table 1. Both from Fig. 2 and Table 1, it can be seen that in the majority of cases, each level is represented as an interval, not just a coordinate point, and these intervals can overlap. Also, significant differences can exist between different doctors.

**Table 1:** An extract of the dataset, for each severity level the min and max values are shown, while the representative point, a scalar value ∈ [0, 100] for each level, is not shown for brevity

*Absent*	*Mild*	*Moderate*	*Severe*	*Extreme*
3–20	23–40	39–55	56–76	83–100
0–18	18–36	37–58	61–81	82–100
2–15	17–37	39–61	63–83	84–97
23–58	44–78	55–93	60–91	71–97
0–9	10–30	30–53	54–77	78–100
7–7	30–30	56–56	67–67	95–95

#### Second data collection: quantitative meaning for potential patients

In addition to the doctors of IRCCS Orthopedic Institute Galeazzi (*N*_*doctors*_=31), the *doctor* sample of our data, we also involved the students enrolled in a computer science bachelor degree class in the 2018/2019 academic year and asked them to involve other potential respondents among their contacts (*N*_*patients*_=1,152); students were given extra credits for participating in the survey and their responses provided the *laypeople* (seen as potential *patients*) sample in this study.

Students were asked to complete a questionnaire similar to the doctor’s, as in the previous section. We then computed the Centroids of the IED (CoIED) for each level (that is, IEDs) in both strata. We also calculated the median, as the data appeared to be affected by noise and dirtiness and thus a more robust central tendency indicator would be more useful, RP of each level, for both doctors and patients.

#### Third data collection: qualitative meaning

Lastly, in order to collect data on the perception of the qualitative meaning of each category, we administered a short questionnaire to the students enrolled in the same computer science class in the following year and their acquaintances. For each questionnaire, a random value is generated a priori in a range from 1 to 99 with equiprobability. The following question is then asked:

“Imagine that you are a patient, and that you are given a scale from 0 to 100, which is often used to represent your health level in numerical form. Imagine that you want to mark on that scale that your health level today is {100 - *random value generated*}. If you had to express in words the same concept, coherently with this numerical value, what expression would you use between the following?”

The respondent is asked to select which category is the most appropriate for his value, from the list of severity categories from HL7. Users are also optionally asked for their sex and age range. We collected 1,257 responses between student and acquaintances. 265 (21%) answers had to be discarded due to an incomplete submission, meaning only 992 (79%) forms were complete and useful for our purposes. For each value in the numerical scale we had an average of 10 complete answers, with a standard deviation of 3.2. For visualization purposes and to enhance the clarity, we performed a binning of the value with granularity of 3, obtaining 33 different bins.

#### Dataset for regression analysis

In order to perform the regression analysis and test the effects, if any, of the proposed encodings we employed a further dataset. This dataset has been collected from real patients who had undergone joint surgery in IRCCS Orthopedic Institute Galeazzi (IOG), one of the major Italian hospitals specialized in musculoskeletal disorders. Specifically, the dataset contains data about 336 patients, with particular reference to so-called Patient Recorded Outcomes (PROMs), that is data reported and collected by the patients (or the doctors) in the last 3 years. In order to measure the effect of the proposed encodings, we considered in particular as a target feature their improvement (on a physical function score) 6 months after joint surgery.

### Representation of ordinal values using fuzzy sets

Starting from the collected data, we will define different techniques for representing ordinal scale level using fuzzy sets [31] and to transform the obtained fuzzy set representations into scalar (or vector) features, so to implement encodings of ordinal features.

#### From ordinal values to fuzzy sets

We will consider a linguistic variable [26] with values in *V*={*v*_1_,...,*v*_*k*_} (in our specific context, the linguistic variable is *Severity condition* and *V*={*Absent, Mild, Moderate, Severe, Extreme* }). In this section, we give a semantics to each term in *V* by means of a fuzzy set in the universe *U*=[0,100]. The precise fuzzification technique that one can adopt, depends on the type of information specified by the involved respondents; indeed, as described in the “Data collection” section, we asked the respondents two different types of information with respect to the representation of ordinal levels in numeric terms: single numeric values (that is representative points), or whole intervals associated to a given level. In the first case, the fuzzification is straightforward: for each term *v* in *V* and each value *x* in the range [0−100] we simply count how many times *x* has been associated to term *v* as a representative point. In the second case, two approaches can be adopted:

1. An indicator of central tendency of the single intervals (such as the centroid of the interval or its median) can be employed to convert each interval to a single numeric value. These values can then be employed straightforwardly to compute the fuzzy sets for each of the ordinal levels.
2. The whole interval can be used to construct the fuzzy set representation of the ordinal levels. In this case, given an interval *i*=[*l*_*i*_,*u*_*i*_] reported by a respondent, where *l*_*i*_ (resp. *u*_*i*_) is the lower (resp. upper) limit of the interval, each point in *i* is weighted by a factor $w_{i} = \frac {1}{u_{i} - l_{i} + 1}$. Then, for each term *v* and each value *x* we count how many times an interval *i* such that *x*∈*i* has been associated to term *v*, weighed by factor *w*_*i*_. Compared with the above mentioned technique, this second approach has the advantage that the whole interval information is explicitly considered in building the fuzzy set, however it has been noted in [31] that simply applying this technique on the raw data may result in too noisy distributions, hence binning techniques should be employed to reduce the granularity.

As a concluding note, we observe that, irrespective of the fuzzification technique adopted, the resulting fuzzy sets are not required to be *fuzzy numbers* [32].

#### From fuzzy representations to encodings

In order to make the fuzzy representations of the ordinal values, obtained by means of one of the techniques previously describer, usable by machine learning algorithms, we need to perform another transformation in order to map the informative but unstructured fuzzy set representation into standard scalar-valued (or vector-valued) features, in a manner which is similar to the traditional *defuzzification* step [33]. To this end, we will describe three different approaches, two of which produce single scalar-valued encodings and one which results in a vector-valued encoding. Let *v* be an ordinal term and *μ*_*v*_:[0,100]↦[0,1] the respective fuzzy set encoding. As regards the first approach, that we call *Centroids of the Interval Extreme Distribution* (CoIED) and is akin to the standard *center of gravity* defuzzification method [33], we simply compute the centroid of the membership function *μ*_*v*_, that is:

1 $$ CoIED(v) = \frac{1}{\sum_{x \in [0,100]} \mu_{v}(x)}\sum\limits_{x \in [0,100]} x*\mu_{v}(x)  $$

Notice that this approach produces the same value for each instance of the *v* label and thus, if the centroids are order-preserving (that is *v*_1_≤*v*_2_ ⇒ *CoIED*(*v*_1_)≤*CoIED*(*v*_2_)) this method always preserves the ordinality of the labels.

The second approach that we describe, and that we call *Weighted Sampling*, is based on a sampling method, similar to Monte Carlo approaches [34] and the sampling defuzzification techniques which can be employed for generalized fuzzy sets [35]. Given the membership function *μ*_*v*_ of an ordinal term *v*, a probability distribution is computed as $p_{v}(x) = \frac {\mu _{v}(x)}{\sum _{y} \mu _{v}(y)}$. Then uniformly across the dataset a value *x* is sampled randomly according to *p*_*v*_(*x*) and each occurrence of *v* is mapped to *x*. Notice that, contrary to the CoIED method, this method can reverse or otherwise change the ordinality of the labels as it may happen that even if *v*_1_≤*v*_2_, for a given row, two values *x*_1_,*x*_2_ are sampled (respectively, from $p_{v_{1}}$ and $p_{v_{2}}$) such that *x*_2_≤*x*_1_.

The third approach, which we call *Membership*, results in a vector-valued encoding and is based on a two-step method. Firstly, given a term *v*, the numeric value *x*_*v*_ which is most representative of it is selected, that is *x*_*v*_=*argmax*_*x*∈[0,100]_*μ*_*v*_(*x*). Then *x*_*v*_ is mapped to the vector of its membership values in the different level-specific fuzzy sets, that is:

2 $$ Membership(v) = \left\langle \mu_{v_{1}}(x_{v}),..., \mu_{v_{k}}(x_{v}) \right\rangle  $$

where, respectively, $\mu _{v_{i}}$ is the membership function associated to the ordinal term *v*_*i*_∈*V*. It is easy to observe that this approach consists of a generalization of one-hot or rank-hot encodings which takes in consideration the inherent vagueness of the underlying ordinal scale: indeed, if the fuzzy sets of the different terms are completely disjoint (that is there does not exists *x*∈[0,100] and *v*_1_,*v*_2_∈*V* such that *v*_1_,*v*_2_≥0) then the result of the membership encoding is equivalent to the above mentioned encodings.

### Ordinal data in machine learning

The fuzzy set representations obtained with the quantitative data collection allow us to address two research questions. First: do doctors and potential patients perceive severity levels differently (on an equivalent 100-scale)? On the other hand, the resulting representations were used to address a second research question: does a user-centered encoding improve the validity of machine learning models on some regression tasks?

To this latter aim, we have compared the performance of 4 common machine learning models, namely Random Forests (RF) [36] and Support Vector Regressor (SVR) [37], whose performance is generally recognized as the best one in data-driven predictive tasks [38], and the *k*-Nearest Neighbour (*k*-NN) [39] and Least Absolute Shrinkage and Selection Operator (LASSO) [40] ones. These regression models were trained on the same dataset whereas, in one case, ordinal values had been encoded traditionally (that is, 0, 1, 2, 3, 4 respectively), and in the other we had applied the CoIED, Weighted Sampling and Membership encodings.

The regression predictive modeling was based on a set of 15 features (namely gender, age, type of intervention, 3 continuous scores and 9 ordinal features, which were all filled in by patients in pre-operative PROMs questionnaires) to predict the functional improvement 6 months after joint surgery, the models were compared with respect to the *Mean Absolute Error* (MAE) metric and *coefficient of determination* (R2). Comparisons among models were performed on the basis of the confidence intervals on 5-fold nested cross validation. In order to account for the randomness in the Weighted Sampling approach, for that encoding only we repeated the process 10 times and calculated average performances.

## Results

In this section we briefly report the results of the statistical procedures conducted in our studies.

### Visualization of quantitative meaning: differences between doctor and patient’s perception

**Fig. 4:**
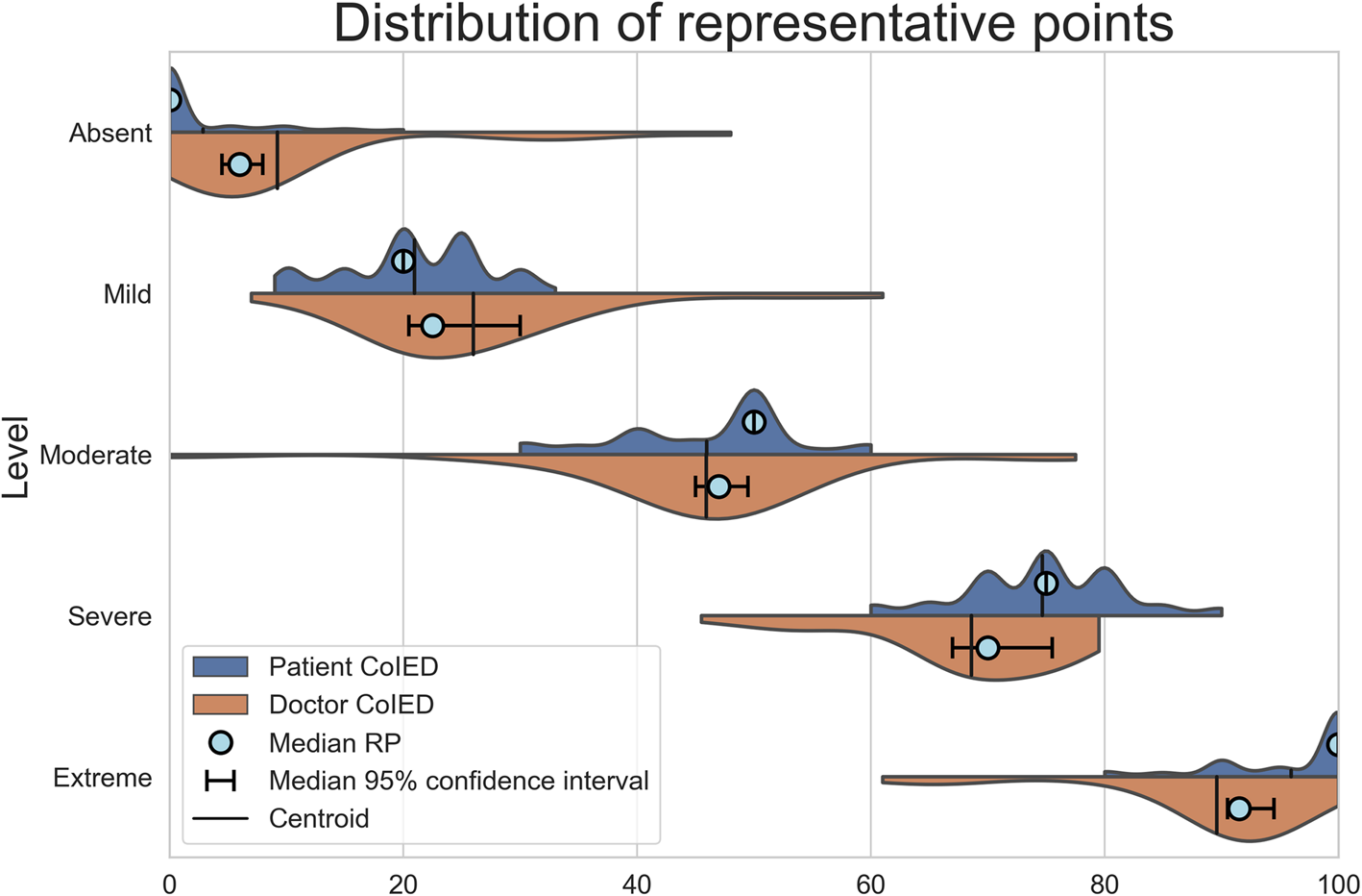
Violin plots of the IEDs for each severity level (red for doctors, *N*_*doctorIED*_=62; blue for patients, *N*_*patientIED*_=[1970, 2155, 1971, 1944, 1670], respectively). The CoIED are indicated as a vertical lines in the violin plots. Small circles indicate the median RPs for each level and stratum (doctors *N*_*doctors*_=31, patients *N*_*patients*_=1,152)

We performed a Kolmogorov-Smirnov test [41] to compare the shapes of the IEDs of doctors and laypeople (Fig. 4). We decided to employ the Kolmogorov-Smirnov test, in place of other goodness of fit such as the Cucconi test or the Anderson-Darling test, as it provides a conservative test for equality of distributions [42] with good quality implementations in standard statistical packages. We found a statistically significant difference in regard to the *Absent* condition and the two highest severity levels (*Absent*, P <0.001, *Severe*, P=0.038 and *Extreme*, P=0.021), while for the other levels the difference was not found significant, although the *p*-values are quite low (*Mild*, P=0.067 and *Moderate*, P=0.145).

We performed a Mann-Whitney *U* test [43] to compare the mean ranks of the patients IEDs (as a sort of hypothetical testing on the equality of their centroids, Table 2) and found significant differences in regard to *Absent*, *Severe* and *Extreme* (P <0.001 in all cases), while differences were not significant for the *Mild* and *Moderate* levels (P=0.425 and 0.105, respectively). We decided to adopt the above test, instead of the Student’s *t*-test, because the main assumptions of this latter did not hold true, and because the Mann-Whitney test is more efficient than the t-test for non-normally distributed data, as well is generally less susceptible to outliers [44].

**Table 2:** Findings from the user study on the perceptions (expressed in terms of CoIEDs and RPs) by doctors and laypeople of illness severity levels. Significance levels are computed through the Mann-Whitney *U* test

Level			Doctor	Patient	Diff
	Doctor	Patient	RP	RP	Doctor
	CoIED	CoIED	median	median	vs
	95% CI	95% CI	95% CI	95% CI	Patient
*Absent*	[4.74, 13.7]	[12.9, 14.6]	[4.5, 8.0]	[0.0, 0.0]	***
*Mild*	[22.2, 29.6]	[25.8, 27.3]	[20.5, 30.0]	[20.0, 20.0]	NS
*Moderate*	[42.6, 50.7]	[40.4, 42.2]	[45.0, 49.5]	[50.0, 50.0]	NS
*Severe*	[63.5, 71.5]	[56.83, 59.15]	[67.0, 75.5]	[75.0, 75.0]	***
*Extreme*	[83.5, 92.5]	[69.12, 72.51]	[90.5, 94.5]	[99.0, 100.0]	***

We also performed a Mann-Whitney *U* test to compare the mean ranks of the RP distributions and found the same significant differences, in regard to *Absent*, *Severe* and *Extreme* (P <0.001 in all cases), while differences were not significant for *Mild* and *Moderate* (P=0.425 and 0.105, respectively).

### Visualization of qualitative meaning

**Fig. 5:**
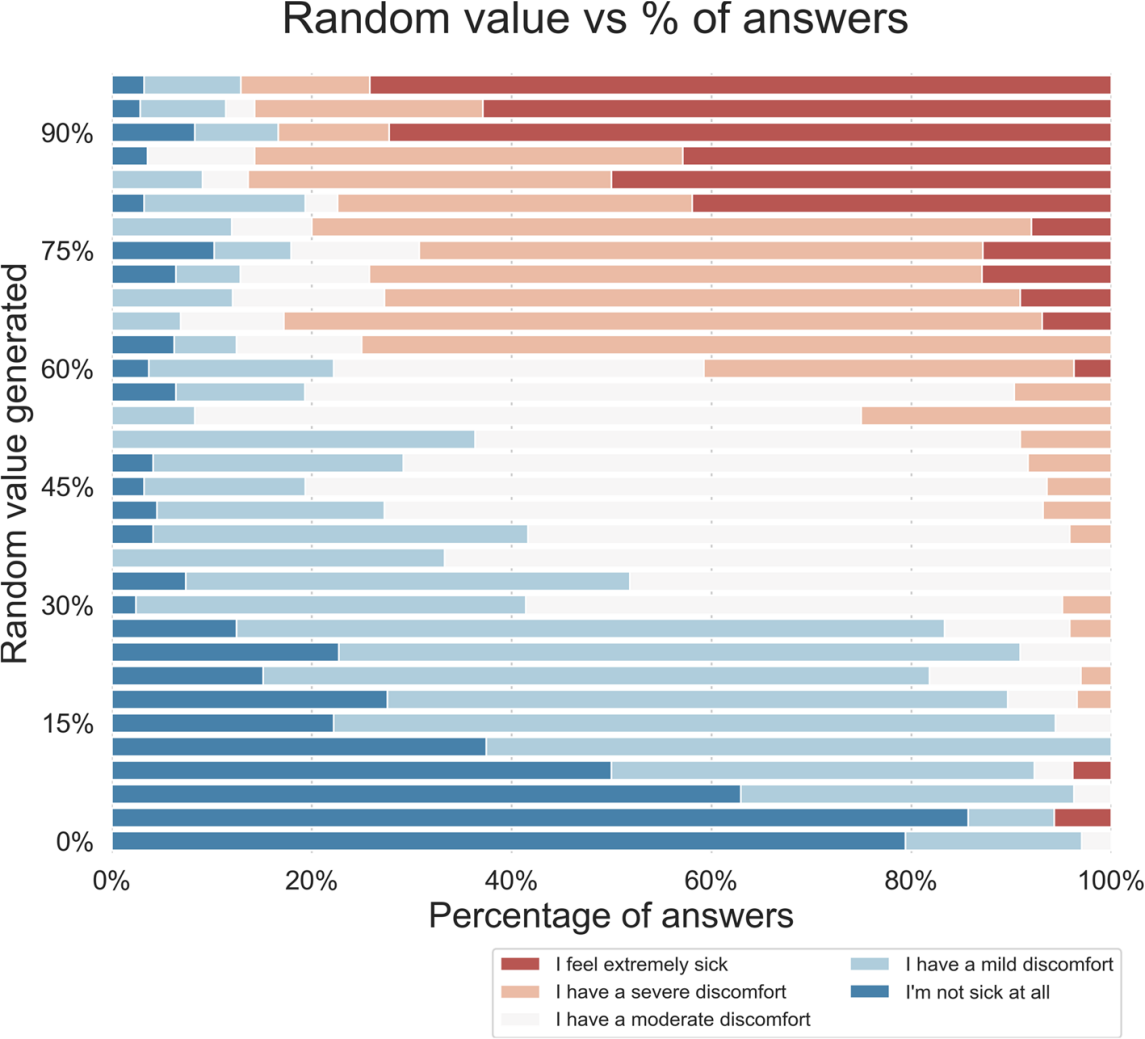
The stacked bar charts indicate, for each bin, the percentage of respondents for each linguistic label. The original labels were in Italian, as shown in the legend, but they can be directly translated to the already discussed HL7 labels

We also investigated the inverse mapping, that is, how respondents mapped precise numerical values to ordinal labels from the Health Level 7 (HL7) terminology. A visualization of this mapping in terms of a stacked barchart is shown in Fig. 5.

**Fig. 6:**
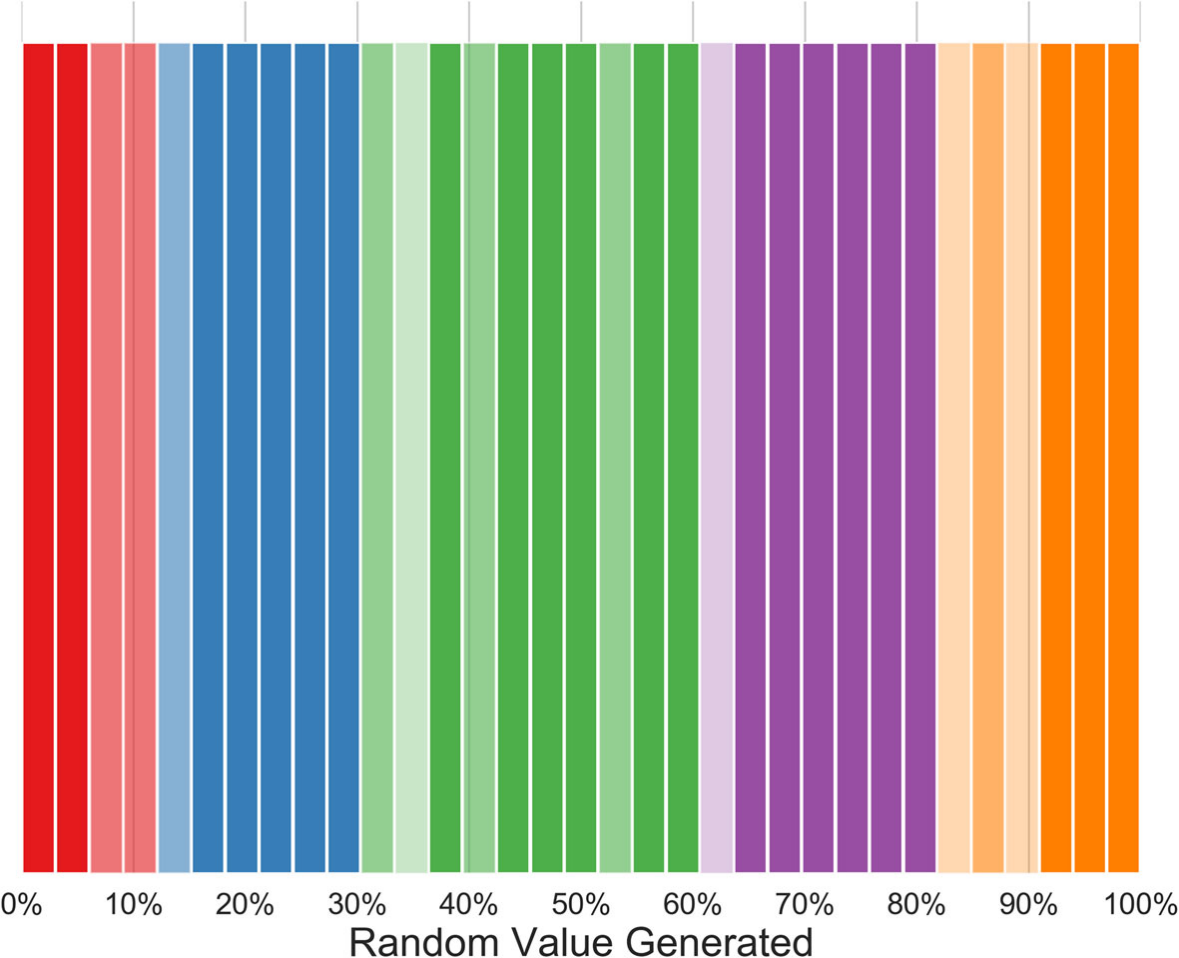
These bar charts indicate, for each bin, the most common variable chosen by respondents and its prevalence

Another way of visualizing this mapping is shown in Fig. 6. The hue represents the most common variable (red for *Absent*, blue for *Mild*, green for *Moderate*, purple for *Severe*, orange for *Extreme*), while transparency represent the prevalence: very light for superiority (mode), medium for majority (prevalence of the most common class >50*%*), opaque for statistical majority (*p*-value <0.05). Statistical majority has been calculated by the means of a *χ*^2^ test between the most common class and the second most common.

### Results of proposed ordinal representations in machine learning

**Fig. 7:**
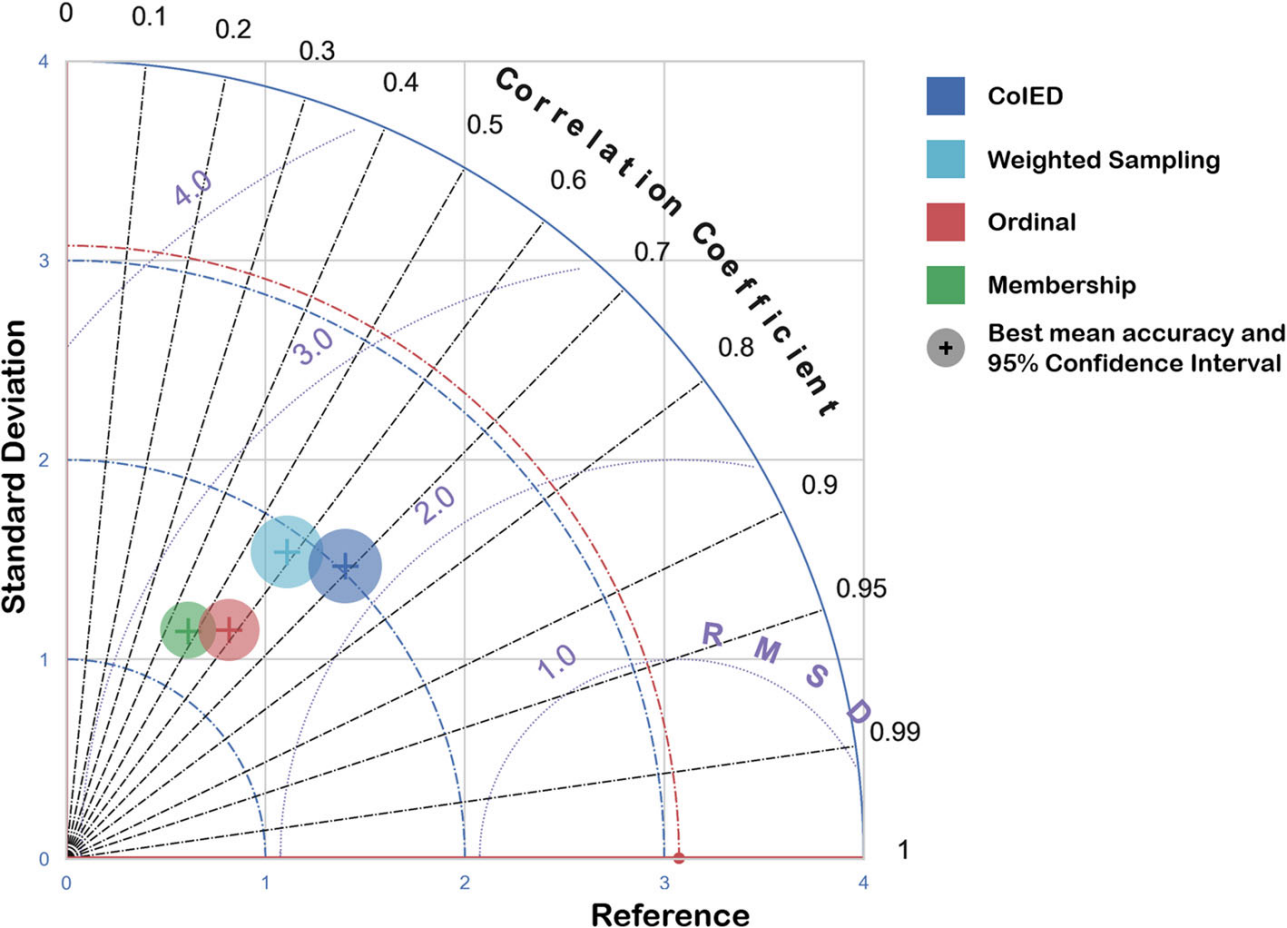
Taylor diagram of the models’ skills. Each point on the diagram indicates the mean performance of all model in a specific representation, with the circle indicating the RMSD 95% confidence interval. RMSD indicates the Root Mean Square Deviation (the lower the better, with 0 denoting a perfect fit to the data). If models denoted with the same glyphs get closer to the inner circle of RMSD and move downward in the diagram (that means that their predictions are more correlated with the true value), then their performance improves and gets better. Red glyphs indicate models with ordinal encodings, while blue glyphs the models with CoIED encodings

In Fig. 7 and Tables 3 and 4 we show the results of the comparative regression analysis, after having trained 4 common models on the dataset discussed in the “Methods” section, in order to predict their improvement (on a physical function score) 6 months after joint surgery.

**Table 3:** The regression performance of the 4 machine learning models considered in the comparative study in terms of Mean Absolute Error (MAE) and related confidence intervals (CIs, at a 95% Confidence Level): the lower the value, the better the performance. The first column presents the CIs of the MAE of models with the ordinal encoding; the second column the same accuracy indicators for the CoIED encoding

	**Ordinal**	CoIED	Membership	Weighted Sampling
RF	[1.458, 1.89]	[1.459, 1.89]	[1.467, 1.861]	[1.688, 1.825]
*k*-NN	[2.012, 2.277]	**[1.503, 1.813]**	[2.078, 2.321]	**[1.731, 1.854]**
LASSO	[1.586, 1.863]	[1.474, 1.736]	[2.121, 2.367]	[1.769, 1.902]
SVR (RBF kernel)	[1.985, 2.312]	**[1.268, 1.736]**	[2.047, 2.373]	**[1.654, 1.829]**

**Table 4:** The regression performance of the 4 machine learning models considered in the comparative study in terms of coefficient of determination (R2) and related confidence intervals (CIs, at a 95% Confidence Level): the higher the value, the better the performance. The first column presents the CIs of the R2 of models with the ordinal encoding; the second column the same accuracy indicators for the CoIED encoding

	**Ordinal**	CoIED	Membership	Weighted Sampling
RF	[0.275, 0.581]	[0.275, 0.58]	[0.291, 0.602]	[0.338, 0.435]
*k*-NN	[0.043, 0.275]	**[0.333, 0.567]**	[0.006, 0.229]	**[0.339, 0.426]**
LASSO	[0.324, 0.545]	[0.364, 0.637]	[0.0, 0.198]	[0.312, 0.416]
SVR (RBF kernel)	[0.017, 0.296]	[0.265, 0.665]	[0.01, 0.26]	**[0.303, 0.427]**

## Discussion

This paper addresses the fuzzification of a common terminology, which is also adopted by the Health Level 7 (HL7) framework in the digital health domain, that characterises health conditions, the appearance of medical signs and other expressions of medical relevance. We show how these are perceived by either the medical doctors or the patients themselves (for instance, in the so called Patient Reported Outcome Measures [19]) and the usage of these fuzzy representations to implement knowledge-based encodings to be used by machine learning algorithms.

### Perception of HL7 terminology

As regards the perception of these terminologies for the two different respondent groups, as highlighted in the “Results” section, we found a statistically significant difference between the distributions obtained for the respondent groups. In particular, we found that patients tend to overestimate the severity of illness, when this is either serious or absent. We can conjecture that differences in the higher part of severity spectrum could be related to the fact that laypeople experience illness in the first person, and hence see it as under a magnifying glass, while doctors have had experience of a much wider range of conditions, relatively few extremely serious and therefore can often scale the assessment lower than patients. By a weaker conjecture, we could see differences in the lower end of the scale as effect of a sort of suppression of the idea to be ill and fear of disease, that induces underestimating light symptoms. These findings, which confirm and are supported by similar findings in the clinical literature [45, 46], have relevant implications, especially as regards their potential impact on machine learning and Artificial Intelligence systems. Indeed, these observations draw attention to the importance of carefully considering the source of data (that is who annotated a specific ordinal value) as the underlying meaning of the same label, even from a standardized terminology as in the case that we considered, could be strongly dependent on who produced the said label. This means that using labels as univocal tokens in advanced statistical techniques, like the ones employed in machine/statistical learning and in the definition of predictive models, can be harmful. The same patient could be associated with a *Mild* label by a doctor, and a *Severe* label by another doctor, and this even if either doctors intend to characterize the very same condition, which could be represented by the same numerical value on a 0-100 continuum. This observation regards the phenomenon of inter-rater reliability that, although widely known in the medical ambit [47], is still little known and considered in most of the fields of applied computer science [3, 48]. For these reasons we argue that any method for properly representing ordinal scales in numerical terms should be grounded on an empirical and human-centered approach, that is, on the subjective perceptions of domain experts for whom the ordinal categories to be fuzzified are meaningful according to the context, right in virtue of their descriptive power and despite their ambiguity. It is noteworthy to say the fuzzification methods proposed and discussed in this paper have been applied to the traditional 5-item severity terminology only as a proof of the concept: we chose this terminology because it is common to many health conditions, used in most medical specialties, and it has also been recently adopted by the HL7 standard developing organization and hence it is nowadays widespread in most digital health applications. However, these fuzzification methods can be applied to *any* ordinal terminology, and not only to those specific of the medical domain.

A potential limitation with respect to this first part of the study, regards the fact that some respondents contacted us after doing the CAWI to warn us that they had found it difficult to move the cursors of the range slider controls on mobile and multi-touch devices like smart phones and tablets. Although we did not collect information on the device used during the CAWI, we can consider that several people could have tried to fill in questionnaire from their smart phones: this could account for some of the “dirtiness” we detected in the original data set (like improbable interval extremes and empty cells). In any case, to our knowledge no study has so far involved more than thirty domain experts to have them represent the quantitative “meaning” (onto a numerical 0-100 range) for the ordinal categories they use in their reports and records on a daily basis.

### Machine learning with ordinal encodings

As regards our second research question, that is investigating the effects of the proposed encodings on the performance of the machine learning models, we recall Table 3: as the reader can easily see, the best performing method (in terms of average MAE) is the SVR algorithm with the CoIED encoding. When considering the confidence intervals, the SVR with CoIED encoding is not significantly better than other models on the same representation (RF, LASSO with Ordinal encoding, all algorithms with the CoIED encodings, RF with the Weighted Sampling encoding and SVR with the Membership encoding) however it has both a smaller lower bound and one of the smallest interval widths. In general, all algorithms except RF obtained a better performance using the CoIED encoding and in particular they were statistically significant for both *k*-NN and SVR. This suggest that, at least for specific model classes, the usage of user-informed encodings can significantly improve the predictive performance. Interestingly, the performance of RF using the Ordinal and CoIED encoding were almost exactly equivalent, the explanation for such a behavior resides in the specifics of the RF training algorithm [49]. Indeed, the construction of the Regression Trees embedded in the Random Forests requires the determination of threshold levels on the features and does not take in consideration the metric distance between the values of a feature but only their ordinality: this means that every feature transformation which is order-preserving, such as the CoIED encoding, results in the same exact trees.

As regards the Membership encoding, there were no statistically significant differences with the Ordinal encoding except for the LASSO algorithm, for which the Membership method had worse performance than the traditional Ordinal encoding.

As regards the LASSO algorithm, a possible explanation of the observed behavior is not completely straightforward. A possible explanation may consists in the fact that the Membership encoding replaces a single feature with a group of features which are mutually related, while this relationship is not taken in consideration when train the LASSO model. In this sense, a group LASSO [50] or sparse group LASSO [51] could be an appropriate choice to properly take into consideration the relations and constraints between the level features introduced by the Membership encoding.

Interestingly, the Weighted Sampling encoding was found to be significantly better than the Ordinal Encoding for *k*-NN and SVR, although generally the CoIED encoding resulted in better average performance. This observation is especially interesting as we did not consider averaging techniques during model training, having just performed multiple samplings for performance evaluation. This suggests that further research should consider the combination of the Weighted Sampling encoding with probabilistic ensembling techniques [52] to assess if these could result in robust and effective methods.

This second part of our study has some limitations, mainly due to its exploratory nature. First, we are aware that performances, as we previously discussed in the case of Random Forests and the CoIED encoding, can vary depending on the match between different encodings, model families, and specific tasks. Even assuming that our encoding is more valid (that is truthful) than the traditional one, for many practical tasks the order information (hence, the Ordinal Encoding) can be as much predictive as the finer-grained one provided by a user-informed one. Although we adopted an approach similar to that applied in [53], we recognize that considering only one task could not be sufficient to draw definitive recommendations. That notwithstanding, we emphasize that we considered a regression task with actual prognostic value that is based on real-world PROMS and clinical data, and that has been integrated in a decision support system currently experimented in a large Orthopedic hospital with promising results.

We are also aware that the observed improvements, while in specific cases statistically significant, are relatively small. That notwithstanding, it is known that significant differences could be associated also to confidence intervals that overlap slightly [54], so our findings must be considered conservative; and most notably all the MAEs observed are lower than the *minimum clinically important difference* values found for the prognostic task at hand [55] (which are at least almost twice as big, if not much bigger).

Furthermore, we are aware that in the specialist literature some methods to encode ordinal variables in numerical terms exist (for instance, *rologit* [56]). For this reason, our future work will be devoted to integrate the knowledge about the user perceptions into these methods to achieve a good compromise between validity and generalization. Also a further validation of the incremental advantage due to the user-informed encoding on different predictive tasks is due.

## Conclusion

In this paper we have provided elements to consider fuzzification as a convenient way to convert single ordinal labels, which are the representation of choice of many predictive models, into numbers by the means of a user-informed approach.

The advantage of this approach lies in the fact just mentioned above: the mapping is made on the basis of the perceptions of a heterogeneous sample of domain experts, in our case, clinicians. If perceptions are collected from the experts who annotated a ground truth data set, this mapping could optimally represent the implicit meaning that group of people, as a collective, attach to the annotation labels, and hence to the classes the machine learning have to work with. Even if the perceptions are not collected from the same group of people involved in the observations and the annotations, the opportune selection of the sample (for instance,through stratified random sampling) could guarantee a certain degree of representativeness and bring forth reasonable and meaningful mappings. We also observed that significant differences may exist in the representations provided by different user groups and argued that these should be taken into proper consideration when working with this type of information, as otherwise using naive encodings could be harmful: leading to noisy or wrong predictions or, perhaps even worse, deceitful or ill-founded conclusions.

We then showed how these novel user-based encoding techniques, and more specifically the CoIED encoding, could profitably be used to enhance the performance of standard classes of machine learning models. We also suggested potential areas of improvements and future research with respect the other two proposed encoding techniques.

In conclusion, we believe this paper contributes to the research line that, within the more general field of machine learning in medicine, aims to embed user-derived knowledge into feature engineering tasks (for instance, [31]), especially in regard to the encoding of ordinal features, which are very common in medical data sets, to improve the validity of predictions and of the data considered for medical decision making.

Our future work will be devoted to integrate the knowledge about the user perceptions into other methods to achieve a good compromise between validity and generalization. Also a further validation of the incremental advantage due to the user-informed encoding on different predictive tasks is due.

## Data Availability

The user questionnaire data generated during the current study are available in the *BMC2020 Public Dataset* repository, located at https://github.com/AndreaSeveso/BMC-2020-Public-Dataset. On the other hand, patient data that support the findings of the machine learning results are property of IRCCS Orthopedic Institute Galeazzi, but restrictions apply to the availability of these data, which were used under license for the current study, and so are not publicly available. Data are however available from the authors upon reasonable request and with permission of the above Institute.

## Abbreviations

CAWI: Computer-Assisted-Web-self-Interview
CoIED: Center of Interval Extreme Distribution
FHIR: Fast Healthcare Interoperability Resources
HL7: Health Level 7
IOG: Orthopedic Institute Galeazzi
IED: Interval Extreme Distribution
IRCCS: Scientific Institute for Research, Hospitalization and Healthcare
*k*-NN: *k*-Nearest Neighbour
LASSO: Least Absolute Shrinkage and Selection Operator
MAE: Mean Absolute Error
PROMS: Patient Reported Outcome Measures
RF: Random Forests
RP: Representative Point
SVR: Support Vector Regressor
VAS: Visual Analogue Scale
